# Precision Preventive Medicine of Relapse in Smoking Cessation: Can MRI Inform the Search of Intermediate Phenotypes?

**DOI:** 10.3390/biology11010035

**Published:** 2021-12-27

**Authors:** Yolaine Rabat, Sandra Chanraud, Majd Abdallah, Igor Sibon, Sylvie Berthoz

**Affiliations:** 1 University Bordeaux, CNRS, EPHE, INCIA, UMR 5287, 33000 Bordeaux, France; yolaine.rabat@u-bordeaux.fr (Y.R.); majd.abdallah@inria.fr (M.A.); igor.sibon@chu-bordeaux.fr (I.S.); 2 EPHE-PSL University, 75014 Paris, France; sandra.chanraud@u-bordeaux.fr; 3 Stroke Unit, Department of Neurology, CHU Bordeaux, 33000 Bordeaux, France; 4 Department of Psychiatry for Adolescents and Young Adults, Institut Mutualiste Montsouris, 75014 Paris, France

**Keywords:** smoking-cessation, addiction, MRI, neuro-biomarkers, insula, relapse

## Abstract

**Simple Summary:**

Addiction to tobacco is a serious health and economical problem because it is one of the most addictive and the most consumed substance in the world. Although well documented, and despite the desire of numerous smokers to quit, maintenance of abstinence is a daily challenge for most of them. The heterogeneity in achieving this maintenance raises the question of potential differences in brain reactivity. An emerging field of research has been interested in brain markers helping to identify individuals who are the most likely to relapse. Using brain imaging techniques such as Magnetic Resonance Imaging (MRI), one can hope it will be possible to offer tailored care for each patient.

**Abstract:**

Chronic tobacco smoking remains a major health problem worldwide. Numerous smokers wish to quit but most fail, even if they are helped. The possibility of identifying neuro-biomarkers in smokers at high risk of relapse could be of incredible progress toward personalized prevention therapy. Our aim is to provide a scoping review of this research topic in the field of Magnetic Resonance Imaging (MRI) and to review the studies that investigated if MRI defined markers predicted smoking cessation treatment outcome (abstainers versus relapsers). Based on the available literature, a meta-analysis could not be conducted. We thus provide an overview of the results obtained and take stock of methodological issues that will need to be addressed to pave the way toward precision medicine. Based on the most consistent findings, we discuss the pivotal role of the insula in light of the most recent neurocognitive models of addiction.

## 1. Introduction

Chronic tobacco smoking is one of the main risk factors of cancers and cardio-vascular diseases and contributes to the death of more than 8 million people every year [1,2]. Hence, it remains a major public health problem with huge economic consequences worldwide. Despite numerous public health campaigns about the deleterious effects of smoking, the recurrent increase of tobacco taxation and the ongoing development of different treatments, few smokers achieve abstinence. Numerically, while most smokers declare a willingness to quit, less than half of them will try, and only 5% will remain abstinent without any help at 6 months, a rate that will increase to about 50% using nicotine replacement therapies (NRT) and/or pharmacological treatment [3,4].

One critical question is, therefore, why is it so difficult for smokers to quit? Numerous factors with potential interactive effects have been associated with a higher risk of treatment failure or relapse, suggesting that a bio-psycho-social approach must be adopted to guide clinical practice [5].

First, at the psychological level, personality characteristics related to poor inhibition skills, such as sensation-seeking and impulsivity, or to increased hedonic sensitivity, such as reward dependence, are thought to impact the cessation attempt. Moreover, in line with the self-medication theory, smoking is commonly used as a coping strategy, to reduce internal stresses or feelings of psychological distress. Relying on such a mood regulation strategy could dampen the motivation to quit [6].

Second, at the behavioral level, routines in user behavior (such as lighting and holding a cigarette) and environmental cues (such as cigarette stores or social situations) might automatically launch the urge to smoke (i.e., the experience of craving [7]), which relates to relapse. The greater the number of years of smoking and/or the number of cigarettes smoked, the more difficult it is for smokers to quit.

Moreover, these psychosocial factors are supported by brain molecular changes. At the cerebral level, nicotine is one of the most addictive drugs and modulates the mesolimbic reward pathways [8]. Notably, each cigarette smoked triggers the release of an important quantity of dopamine in the nucleus accumbens that mediates the rewarding and pleasure effect of nicotine, which in turn reinforces tobacco dependence. However, the magnitude of this effect and its consequences on global brain functioning is different between subjects, depending on the individual genetic background encompassing a large setting of genes involved in drug metabolism, susceptibility to addictive behaviors or susceptibility to psychiatric diseases, but also changes in the brain structure and function [9].

Such consequences on the brain can be approached by neuroimaging studies, with already existing reviews demonstrating that the brain is structurally and functionally modified by chronic tobacco smoking [10,11]. Nowadays, there is hope that neuroimaging studies will also help to establish not only ‘where’ and ‘how’ in the brain smoking cessation treatments work, but also why their effectiveness varies between patients. Indeed, from a clinical perspective, it could improve, or even outperform, the current outcome predictive factors of who will be a responder or not to smoking cessation treatment, and thus use these biomarkers to tailor the treatment strategies. Until now, the major predictors used in clinical practice are the number of cigarettes smoked by days and/or a scale of dependence, such as the Fagerstrom Test for Nicotine Dependence (FTND) [12].

The idea that a given treatment should be provided according to the individual susceptibility to respond or not to such treatment is the cornerstone of the precision medicine concept, which “does not literally mean the creation of drugs or medical devices that are unique to a patient, but rather the ability to classify subpopulations that differ in their susceptibility to a particular disease, in the biology and/or prognosis of those diseases they may develop, or in their response to a specific treatment” (see Appendix E [13]). In this framework, the suggestion of using neurobiomarkers for predicting smoking cessation success is not new and was further supported by the results of Naqvi et al. in 2007 who reported the spontaneous cessation of smoking after a brutal acute insular brain insult in a cohort of 69 smokers [14]. However, due to the traumatic nature of stroke, these results are difficult to transpose to all the persons willing to stop smoking. Therefore, examining brain structures as well as cerebral anatomical and functional networks among treatment-seeking smokers, or nicotine-dependent individuals willing to quit, is important to understand which of these markers can be used to predict the success or failure of smoking cessation interventions.

The purpose of this article is thus to present an overview of the progress made in this field of research and what would remain to be improved for offering, in a longer term, more personalized programs.

## 2. Materials and Methods

We searched PubMed from MEDLINE for original articles for the following indication terms: (nicotine OR tobacco OR smoke*) AND (abstinence* OR cessation OR relapse*) AND (fMRI OR MRI OR “real-time fMRI” OR neuroimaging OR neurobiomarker). Papers had to be on humans, using brain magnetic resonance imaging (MRI), published in the English language between January 2007 (publication date of the above-mentioned seminal paper relating the beneficial effect of brain injury on successful smoking cessation) and December 2021 and restrained to tobacco consumption. We excluded studies with an endpoint other than comparing abstainers versus relapsers, such as studies interested in the change in smoking habits or tobacco craving or with an abstinence length of less than one week.

For each article, we reported the characteristics of the population, type and duration of therapies, length of abstinence, the proportion of relapse at follow-up, methodologies of brain MRI acquisition, and analysis and the conclusion from the authors.

## 3. Results 

**Figure 1:**
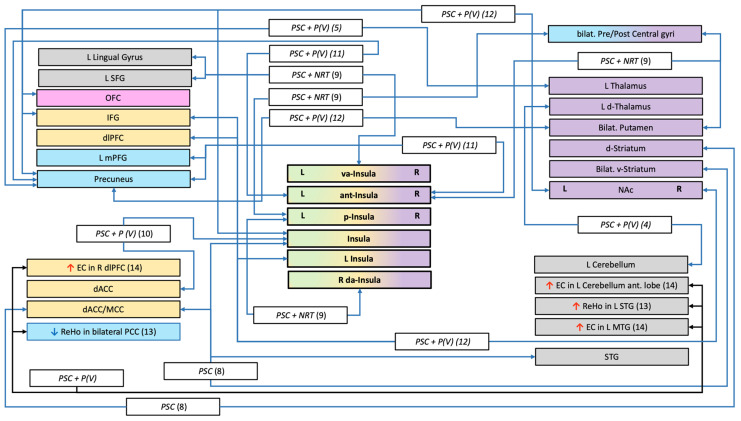
Structures and connections highlighted as precursor to relapse after a quit attempt in resting-state studies. <—> = lower connectivity between structures; ↑ = greater fMRI activation; ↓ = lower fMRI activation; □ white rectangle = treatment use (NRT (Nicotine Replacement Therapy), P (Pharmacotherapy with: V = Varenicline, EVP = selective alpha-7 nicotinic acetylcholine receptor); PCS (Psychological Counselling Session)); □ coloured rectangles = network implicated (Blue = Default Mode Network; Gray = Undetermined network or not part of a specific network; Green = Salience Network; Orange = Executive Control Network; Pink = Reward network; Purple = Habit formation); (n) = number of the study (see Table 1); EC = Eigenvector centrality; ReHo = Regional Homogeneity; Ant = anterior; d = dorsal; dl=dorsolateral; L = Left; v = ventral; va = ventro-anterior; R = Right; m=medial; ACC = Anterior Cingular Cortex; IFG= Inferior Frontal Gyrus; OFC = Orbital Frontal Cortex; NAc= Nucleus accumbens; SFG = Superior Frontal Gyrus.

**Figure 2:**
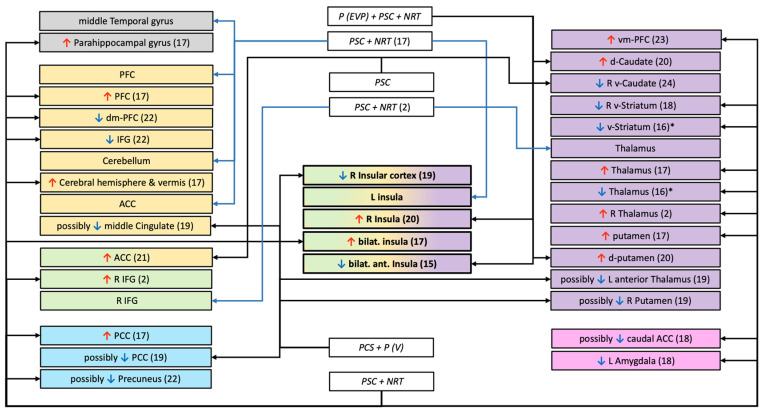
Structures and connections highlighted as precursor to relapse after a quit attempt in task-based studies (drugs-cues, monetary reward, or inhibition tasks). ↑ = greater fMRI activation; ↓ = lower fMRI activation; □ white rectangle = treatment use (NRT (Nicotine Replacement Therapy), P (Pharmacotherapy with: V = Varenicline, EVP = selective alpha-7 nicotinic acetylcholine receptor); PCS (Psychological Counselling Session)); □ coloured rectangles = network implicated (Blue = Default Mode Network; Gray = Undetermined network or not part of a specific network; Green = Salience Network; Orange = Executive Control Network; Pink = Reward network Purple = Habit formation); * = visual inspection of the figure; (n) = number of the study (see Table 1); d = dorsal; dm = dorso-median; L = Left; v = ventral; vm = ventro-median; R = Right; ACC = Anterior Cingular Cortex; IFG = Inferior Frontal Gyrus; PCC = Posterior Cingular Cortex; PFC = Pre Frontal Cortex.

Twenty-four articles meeting our selection criteria were identified. Their main methodological characteristics are summarized in Table 1 (See Supplementary Material for the flowchart). In addition, the MRI findings are presented graphically in Figure 1 and Figure 2.

In all these studies, participants had at least one MRI acquisition before any therapeutic intervention and a predefined quit date. Nevertheless, the time-lapse between MRI acquisition and the beginning of treatment varied across studies. Moreover, therapeutic modalities (type of treatment and duration), as well as the definition of abstinence, were specific to each study. Given this heterogeneity, it was not possible to conduct a meta-analysis and we focused on a descriptive analysis and critical synthesis.

The different studies were first grouped according to the MRI acquisition: anatomical, resting state (RS), and task-based. They were further layered by the type of cessation treatment used: NRT (but independently from the route of administration and dosage), pharmacological treatment (but independently from the drug subtypes and dosage), and psychological counselling sessions (PCS) (but independently from the number of sessions, the qualification of the therapist, and the method used).

### 3.1. Anatomical 

Five studies [15,16,17,18,19] addressed the question of macrostructural anatomical differences underlying the ability to achieve abstinence. They differed in both the type of treatment and the length of abstinence. Three used a whole brain (WB) voxel-based morphometry (VBM [20]) analysis [15,16,18] and the other used different regions of interest (ROIs) [17,19]. The two studies from Froeliger et al. reported differences in grey matter volume (GMV) between abstainers and relapsers, either in different regions or in opposite direction (Table 1). In the three studies using varenicline and PCS, relapse was associated with decreased GMV in the right post-central gyrus, right dorsal striatum, and left OFC in one study [18], and with increased left thalamic GMV in another one [19]. The third study found no association between GMV in insular subregion (TIV corrected) and treatment outcome [17].

Two studies from the same research team investigated if microstructural anatomical alterations are associated with treatment outcome (varenicline + PCS) [21,22]. Using a whole brain approach and comparing 28 abstainers and 38 relapsers, relapse was associated with higher fractional anisotropy in the cerebellum and post-central gyrus [21]. In a further study including as many relapsers but fewer abstainers and an insular ROI, no differences were observed between the two groups [22].

### 3.2. Restings State (RS) 

Functional MRI RS connectivity analyses are based on the demonstration of strong temporal correlations between the spontaneous, intrinsic fluctuations at rest of the BOLD signal (reflecting neuronal activity while people lie still and think of nothing in particular) in different regions. The time-course of this signal from the seed-region is correlated with the BOLD signal time-course in every other voxel in the brain. These communicating regions tend to be engaged simultaneously throughout daily tasks and sustain a communication activity even in resting conditions [23].

Brain networks reconstructed by RS analyses are highly consistent within and between individuals and present high functional and anatomical coherence [24].

The more frequently reported and consistent networks across studies are the Default Mode Network (DMN), the task-positive (in opposition of the DMN), the sensorimotor network, the visual network, the fronto-parietal network (sometimes named the attentional network), and the auditory network.

We identified nine studies that examined if the pre-cessation brain activity at rest would predict the treatment outcome.

In the Sweitzer et al. (2016) study, participants underwent two fMRI sessions before the initiation of a PCS: the first after smoking ad libidum and the second after 24 h of abstinence. These authors used a whole-brain connectivity approach with the ventral and dorsal striatum as seed regions and modelled age, sex, cigarettes smoked per day and session order as covariates. Relapse was independently associated with weaker connectivity after abstinence (relative to satiety) between the bilateral ventral striatum and the insula, the superior temporal gyrus (STG), and the anterior/mid cingulate cortex [25].

In a study combining NRT and PCS, Addicott et al. (2015) focused on insula-based networks and used treatment group and FTND scores as covariates. Relapse was independently associated with weaker functional connectivity between (i) the posterior insula and pre-central gyrus, the post-central gyrus, and bilateral putamen; (ii) the ventral anterior insula and left superior frontal gyrus (SFG) and lingual gyrus; (iii) the right dorso-anterior insula and left posterior insula [26].

The study by Wilcox et al. (2017) that included people treated either by pharmacotherapy or placebo focused on the functional connectivity between six ROIs (Table 1) [27]. Taking the treatment group into account, they found no differences between the relapsers and abstainers. Nonetheless, irrespective of treatment group, lower levels of connectivity between the insula and dorsal anterior cingular cortex (dACC) predicted greater smoking quantity prior the endpoint, and this was the case independently from the FTND score.

In four studies conducted by the same research team, the treatment combined varenicline and PCS [18,19,28,29]. All studies used a whole brain approach but different seed regions. Two studies assessed the association with baseline connectivity only [18,19], and the two others compared post- versus pre-treatment changes in connectivity [28,29]. Relapse was associated with reduced baseline functional connectivity between the thalamus and cerebellum in the Qian et al. (2019) study, and between the thalamus and precuneus in the Wang et al. (2020a) study. During treatment, relapse was associated with decreased connectivity (from before to after the treatment) between the insula and the precuneus as well as the medial frontal gyrus [28], and between the striatum and the insula, orbito-frontal cortex (OFC), dorsolateral PFC, inferior frontal gyrus (IFG) and precuneus [29], while abstainers showed the opposite pattern of changes.

Another voxel-wise method to evaluate local functional connectivity is the measure of regional homogeneity (ReHo), which is defined as the average functional connectivity values of a voxel with its surrounding voxels and reflects how much each region is connected to its neighbors [30]. Using this method, Wang et al. (2017) demonstrated that, among people who received a pharmacological treatment combined to PCS, relapse was associated with a decreased ReHo in the bilateral posterior cingulate cortex (PCC) as well as an increased ReHo in the left STG, independently from baseline smoking and FTND score [31].

Finally, Shen et al. (2017) used a voxel-wise eigenvector centrality (EC) measure to identify key functional brain networks. EC gives information about the wide-reaching influence of a functional area. The eigenvector value is relative to the number of connections to other highly connected areas [32]. In a sample under pharmacological treatment, independently from baseline smoking, relapse was associated with increased EC in the middle frontal gyrus (MFG) and SFG, the left middle temporal gyrus (MTG) and STG, and the left anterior cerebellum [33].

### 3.3. Task-Based Studies

#### 3.3.1. Functional Activation 

Functional activation studies exploit endogenous brain activity for mapping brain responses during periodic and specific cognitive/emotional tasks.

##### Inhibition 

Several models consider that the development and maintenance of addiction is tightly linked to impaired inhibitory control (IC) following exposure to a representation of the substance. We found two articles that examined if the pre-cessation brain reactivity during IC tasks predicted treatment outcome using an ROI approach.

Froeliger et al. (2017) investigated the activation of the right inferior frontal gyrus (IFG), bilateral thalamus, subthalamic nucleus, presupplementary motor area, and left primary motor cortex during a Go/NoGo task with various neutral stimuli. Following a combined treatment of NRT and PCS, relapse was associated with increased activation in the right IFG and thalamus, and this was the case independently from FTND score [15]. Moreover, greater right IFG activity was associated with worse behavioral performances.

Gilman et al. (2018) also used a Go/NoGo task with tobacco-related (versus neutral) images and a ROI approach among people under four different treatments (pharmacotherapy EVP-6124 and/or NRT in addition to PCS). Relapse was associated with decreased activation in the bilateral anterior insula with tobacco-related stimuli NoGo trials independently from FTND score but without taking the treatment group into account [34].

##### Drug Cues or Healthy Messages 

Because people with a substance use disorder are more sensitive to environmental cues specifically related to their drug consumption, drug-cues reactivity paradigms are considered an ideal way to observe engaged pathways of reward- and/or cognitive-control-related regions to predict substance use disorders treatment outcomes [35].

##### Smoking Cues vs. Neutral

Out of the six studies that were identified, all but three [36,37,38] used an ROI approach but different ones.

Three studies included patients treated with NRT combined to PCS [37,39,40]. The McClernon et al. study (2007) involved three fMRI sessions (baseline, quit date, 2–4 weeks following quitting). At baseline, relative to abstainers, relapsers seemed to have lower smoking cue reactivity in the ventral striatum and thalamus. Moreover, there was a Scan session X Stimulus type X Treatment outcome interaction. For relapsers, the differences between responses to smoking and control cues were negligible at all three scans; for abstainers, there was a greater activation to smoking than control cues in the thalamus and ventral striatum before treatment, and this pattern was reversed after treatment. In the Jane et al. study (2010), relapse was associated with greater smoking cue reactivity in the bilateral insula, PCC, parahippocampal gyrus, thalamus, putamen, cerebellar hemispheres and vermis, and prefrontal cortex (PFC). In the most recent study, relapse was associated with lower smoking cue reactivity in the ventral striatum and amygdala independently of the level of craving before and during the scanning session [40].

Two studies were conducted among smokers treated by pharmacotherapy either alone [36] or in combination with NRT [41]. In the study by Janes et al. (2017), relapse was associated with greater reactivity in the right insula and dorsal striatum (dorsolateral putamen and dorsal caudate). Hartwell et al. (2013) designed a more complex paradigm involving two fMRI sessions (baseline and post quit) with two experimental conditions each [36]: while being allowed to experience craving, and conversely when being asked to resist the craving. A whole-brain analysis at baseline revealed no specific brain activation associated with smoking cessation outcome when participants were allowed to crave. Conversely, during craving resistance at baseline, relapse was associated with a lower reactivity in the insular cortex, putamen, anterior thalamus, middle cingulate, and PCC. When contrasting the baseline and post-quit sessions, no differences were found for either the crave or resist conditions among the relapsers, while, for the abstainers, greater activation was found at baseline in the bilateral SFG (extending into the PFC) but in the resist condition only.

Finally, Allenby et al. (2020) also used two fMRI sessions during cue exposure but both at baseline: the first after smoking ad libidum, and the second after 24 h of abstinence. Using a whole-brain analysis, and contrasting the sessions (abstinence versus smoking), relapse was associated with a greater cue reactivity in the ACC in people treated by PCS independently from abstinence-induced changes in craving and withdrawal, sex, age, and baseline smoking [38].

##### Health Messages 

The two studies that investigated the brain reactivity to messages on the negative health consequences of tobacco used a ROI approach in people under NRT combined with PCS [42,43].

Based on the general evidence that tailored interventions are more effective than generic ones, Chua et al. (2011) used audio-visual blocks of personally tailored, untailored and neutral messages. Relapse was associated with decreased activation in the medial PFC, IFG, and possibly in the precuneus following tailored (versus neutral) messages specifically, and independently from number of cigarettes per day at baseline. Owens et al. (2017) focused on the difference between graphic warning labels or text only warning labels and control images. Relapse was associated with greater activation in the ventromedial PFC during graphic warning labels (versus control images), and this was the case beyond the influence of FTND score.

##### Reward 

While smoking is known to be associated with a dysregulation of the reward system, determining if a global (i.e., to multiple rewards, such as monetary and food stimuli) or a domain-specific dysfunction (the substance) predicts the issue of the quit attempt is believed to facilitate the identification of more targeted interventions such as contingency management (i.e., providing monetary incentives for periods of abstinence).

This question was addressed by Sweitzer et al. in the same study sample described above (see section B.Resting state) [44]. Relapse was associated with a decreased activity in the striatum (right ventral caudate) with monetary reward anticipation during abstinence (versus satiety), but not in the smoking anticipation condition. This effect remained after controlling for age, sex, and the number of cigarettes smoked per day, as well as the level of craving during the 24 h of abstinence.

Based on the consistent finding that substance use disorders are associated with poor abilities to delay gratification (e.g., choose 10 euros immediately over 30 euros in 3 months, also called temporal discounting), Grosskopf et al., (2020) examined if post- versus pre-treatment changes in brain activity during a temporal discounting task is associated with treatment outcome. No significant differences were observed between relapsers and abstainers [45].

#### 3.3.2. Functional Connectivity

Cerebral areas’ interactions allow the study of the brain as a network justifying connectivity approach. Using functional imaging data, researchers can investigate not only which individual brain areas are involved in a task, but also how information flows between brain areas [46] and how functional areas change their connectivity to participate in different networks at different times (Smith et al., 2012) or under different behavioral circumstances.

We identified three studies that examined if the pre-cessation task-based functional connectivity (tbFC) predicted treatment outcome.

Two included participants treated by a combination of NRT and PCS [15,37]. Janes et al., (2010) used the presentation of smoking cues (vs neutral ones) and two Independent Component Analysis (ICA) defined ROIs and found that relapse was associated with weaker tbFC between left insula, PFC, ACC, cerebellum, and MTG [37]. Finally, using functionally defined ROIs selected from the data collected during the IC task that has been described above (NoGo versus Go trials, see section C.1.a) and baseline FTND scores as nuisance covariates, Froeliger et al. reported that relapse was associated with a decreased tbFC between the right IFG and the thalamus [15]. Notably, the tbFC mediated the association between behavioral inbibitory control performances and treatment cessation outcome.

## 4. Discussion

Despite the implementation and enforcement of tobacco control policies and the development of different types of interventions, smoking remains a defining challenge in global health with huge individual and economic consequences. Even with the help of treatment, still about one out of two smokers fail to achieve successful abstinence. Tailored strategies allowing the early identification of patients at high risk of relapse are urgently required. Magnetic Resonance Imaging, which is able to capture brain anatomy and activity in targeted areas and networks, might be one of the most interesting tools providing biomarkers for this purpose.

In this overview on the characterization of MRI cerebral biomarkers predictive of smoking cessation treatment outcome, we identified 25 studies including about 1250 patients that investigated this topic over the past 15 years. The fact that we did our research only in PubMed could have reduced our findings. However, as we also included studies that were identified from reference lists, we think this had little impact on our findings. Two main observations can be drawn from these data: (i) the insula, along with its associated networks, is the structure the most consistently associated with the ability to quit smoking, and (ii) the methodology used in these studies is highly heterogeneous, impeding a quantitative synthesis despite the significant number of patients evaluated.

**Figure 3:**
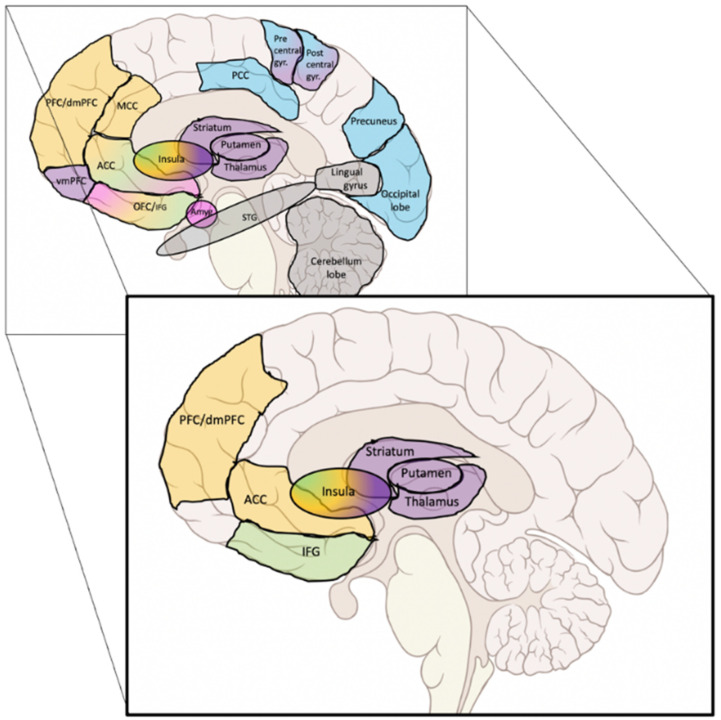
Main results of the structures and networks that predicted treatment outcome. Background rectangle: graphical summary of all the findings. Forefront rectangle: most consistently reported findings. Colours = network (Blue = Default Mode Network; Orange = Executive Control Network; Green = Salience Network; Purple = Habit formation; Pink = Reward network; Gray = Undetermined network or not part of a specific network).

While a meta-analysis could not be practicable, the insula appeared tightly involved in smoking cessation treatment outcomes. This finding reinforces the observation from the seminal work of Naqvi et al. (2007) showing that insular damage increased the likelihood to quit smoking abruptly and easily. This was confirmed and extended by further prospective studies [47] with an impact on longer abstinence [48] together with increased motivation for remaining abstinent [49], less frequent and severe withdrawal symptoms [50], and decreased smoking urges [51]. Notably, as summarized graphically in Figure 3, specific patterns of connectivity of the insula were predictive of treatment outcome. This observation adds to the evidence implicating the insula in tobacco addiction through the dysregulation of a complex network rather than a mere macrostructural insular change.

At the anatomical and histological level, three major subdivisions of the insula have been identified in humans and animals, including the granular insula, located in the posterior dorsal portion, the agranular insula in the anterior ventral portion, and the dysgranular part in the middle portion. Each subdivision has different connectivity features and therefore differs functionally. In humans, the insula has been described as the main node of the saliency network and as an integral hub in mediating dynamic interactions between other large-scale brain networks involved in externally oriented attention and internally oriented cognition [52]. RS fMRI studies have also pointed out its rich connectivity both with other regions and between its functional subdivisions, which may explain how the saliency network participates in so many diverse functions. Interestingly, latency analyses have revealed that insula activity temporally precedes activity in the other nodes of the executive and Default Mode Network (DMN). This new understanding of the insula as a critical node for network switching may explain its implication in substance use disorder. Indeed, it has been proposed that, first, sensory features of interoceptive signals are processed in the posterior insular cortex, then, the corresponding information is passed to the anterior insular cortex where it is remapped by integrating the motivational, emotional, and cognitive inputs from its subcortical and cortical connections and then further translated into a subjective feeling and/or conscious thought. This feeling will, in turn, influence homeostatic goals and approach/avoidance behaviors by connecting to different prefrontal regions.

Besides the insula and prefrontal cortex, our review also points to an important role of the basal ganglia and thalamus in smoking cessation. The ventral striatum has classically been considered to play an important role in addiction through its role in the anticipation and immediate response to rewards [53,54], as well as in abstinence-induced craving [55,56,57]. A recent study showed that acquired damage in the dorsal striatum is associated with an increased likelihood to quit smoking easily and immediately and to remain abstinent [58]. The ventral striatum directly receives the projection of glutamate neurons from the orbitofrontal cortex, while the dorsal striatum interacts with thalamic-cortical circuits that are involved in the planning and execution of motor responses. Because smoking behavior becomes highly automatic in people with smoking addiction, the entire striatum might be a key determinant of addiction and relapse processes. Interestingly, in a prospective lesion study on the combined effects of insula and basal ganglia lesions (versus damage elsewhere) on smoking cessation, lesions including both regions were associated with higher rates of abstinence over a one-year period. Moreover, the proportion of abstainers over the follow-up period was 75% in the group with both basal ganglia and insula lesions, while it was the case for 37% of the group with lesions to the basal ganglia alone [59].

Overall, the findings highlighted in our graphical summary (Figure 3) support recent integrative models of addiction. In the neurocognitive triadic model [60], the insula has been described as a core hub, where the anterior part of the insula converts a bottom-up interoceptive message into a subjective emotional state, which in turn modulates the activity of both a subcortical “impulsive” (i.e., amygdalo-striatal) and a cortical “reflexive” (i.e., prefrontal cortex) systems. In the integrated theoretical model from Naqvi and Bechara [47], the insula is also viewed as a hub, playing a critical role in the balance between a subcortical automatic mode of drug seeking (largely stimulus/cue driven) and a cortical goal-directed one (manifested by cravings and the tendency to relapse), by engaging representations of interoceptive drug effects in order to overcome drug-associated subjective risks and conflicts (such as dealing with negative consequences or competing goals). From a clinical perspective, these authors suggest that the most effective approach would be a two-step treatment, first by promoting the goal-directed mode with interventions that foster awareness/insight (i.e., heightened subjective riskiness of drug use) and second to buffer the insula function with neuromodulatory medications or interventions.

Unfortunately, a relatively high proportion of the studies that have been conducted up to now have methodological weaknesses that preclude strong conclusion(s) that could help to guide clinical management.

First, the definition of abstinence is variable between studies and sometimes mixed up with successful quitting. Because the determinants of early and late relapse are different [61], and as relapses occur most often within the first year following smoking cessation [62], the clinical endpoint should be at least evaluated after this delay to maximize the predictive power of the brain marker.

Second, while a significant proportion of the studies were randomized clinical trials, cessation outcome groups were defined regardless of the type of treatment, and this potentially confounding variable was not systematically accounted for.

Third, there is a need for better phenotyping of the study population. Owing to the cost of this approach, a biomarker designed to be used in patient’s treatment stratification has to improve the prediction of success of the intervention on top of the simplest and/or already known clinical factors. Accordingly, none of the neuroimaging findings should be interpreted without considering the level of impregnation (i.e., the number of cigarettes smoked per day, the number of years of exposure, carbon monoxide level, cotinine, etc…) or the severity of the nicotine addiction (i.e., the level of dependence to the substance, the frequency and intensity of craving episodes and withdrawal symptoms, etc…). Unfortunately, not all the studies from this review used these factors as covariates, which mitigates the interpretation of the results. In addition, because poly-drug use is not only frequent among smokers but also a source of consumption cessation failure as well as a condition that impacts the brain, this parameter should be better documented and considered. In this regard, investigating potential interactions between the patients’ age and addictive profile could be an important issue. In fact, nowadays, we witness in clinical practice that smoking tobacco is increasingly associated with cannabis consumption among the youngest, while alcohol daily consumption is most frequently observed in older patients. Moreover, mood factors and their associated clinical conditions such as depression and anxiety disorders, which are known to be associated with the susceptibility for relapse and with functional brain changes, need to be investigated and modelled. This point might be particularly critical as a recent metanalysis [63] further emphasized a bidirectional link between smoking behavior and depression/anxiety, baseline depression/anxiety being associated with future smoking consumption, while smoking exposure contributed to the occurrence of later depression/anxiety. Besides the dysphoric state, other psychological factors have still not yet received attention in the fMRI literature we reviewed. Other cognitive functions than inhibition and cue-reactivity are considered to play a role in addiction, notably interoceptive awareness and/or metacognitive skills [64,65]. This point is actually raised in the new metacognitive hub model of craving defended by Flaudias et al. [66] who proposed to refine the triadic neurocognitive model of addiction by considering craving as a multidimensional phenomenon instead of a unitary process. These authors argue for a triadic model that includes not only physiological craving (mediated by the interoceptive system), but also automatic craving (mediated by the impulsive system) and cognitive craving (mediated by the reflective system) as well as their interactions and reinterpretation by metacognitive skills (playing the role of a hub between the systems).

Fourth, there are some cerebral potential confounding factors that should be given consideration. With BOLD analysis, two main factors should be more systematically taken into account: baseline perfusion status and the cortical brain volume. Arterial Spin Labeling studies have reported decreased perfusion in multiple brain regions among chronic smokers, including the orbito-frontal cortices and parietal lobules [67]. In addition, several studies have highlighted that smoking constitutes a hypertension risk factor [68], which has been found to reduce cerebrovascular reactivity [69] in brain networks such as the DMN. This chronic perfusion deficit could underlie the smaller total gray matter volume reported in people that ever smoked regularly [70,71]. In addition, as white matter connections are at the foundation of neural networks, functional network analysis should also consider the structural integrity of white matter bundles before drawing any firm conclusion. It is of importance to note that smoking has been associated with a progression of white matter hyperintensities [72,73], as well as with microstructural changes in the normal-appearing white matter [21,70]. Most importantly, these microstructural brain changes have been found to predict smoking cessation outcomes after pharmacological intervention [21,28]. Altogether, these data support the need to assess more systematically brain morphological data as potential confounders in functional neuroimaging results, a methodological precaution that has been ignored in most of the studies.

In addition to the chronic perfusion changes observed in daily smokers, the significant influence of short-term abstinence on regional cerebral blood flow [74] highlights the need to standardize the acquisition of brain MRI in this population, ideally with a first acquisition after overnight abstinence and a second after few cigarettes. Indeed, the physiological changes associated with craving (i.e., transient increase in blood pressure and heart rate) might also influence the regional cerebral blood flow. 

Besides the consideration of these different biomarkers, any metanalysis is also precluded by the heterogeneity in methodologies used to evaluate brain activity. Not only ROI and WB analysis were used, but also different types of images processing that follow the recent advances in brain imaging. While interesting at a pathophysiological level, the replication of former results using the same methods would have been an important first step. Moreover, the different tasks used to evaluate baseline brain networks recruit different brain areas depending on their cognitive and/or emotional load, while the type of stimulation, using visual, auditory or olfactory primary integration might simulate different pathways and could account for the discrepancies observed between studies. In addition, the interpretation of the results has to take into account that participants were treatment-seeking and may not necessarily be generalizable to all smokers. The fact that the participants were volunteers could imply a strong involvement of prefrontal networks (Control Executive Network) that could modulate RS and task-related brain activations networks.

Finally, the main objective of these studies has to be clarified, focusing either on the investigation of the pathophysiological mechanisms of smoking relapse or the identification of individual risk biomarkers that could be used in daily practice, this latter needing the development of individual parameters rather than group analysis.

## 5. Conclusions

Although gaining knowledge on the neural substrates involved in smoking cessation outcomes is crucial to guide treatments, our narrative synthesis of the literature emphasizes that this field of research is still in its infancy, with too little empirical evidence and important methodological heterogeneity. However, it is noteworthy that the findings reported here seem to converge towards a pivotal role of the insula in the modulation of inter-related brain networks. Adequately powered studies, allowing to take into account heterogeneity across treatment-seeking smokers are needed to confirm this suggestion. In addition, if functional connectivity analyses appear the most promising strategy, up to now only static connectivity patterns have been examined. Given that the insula is a well-known hub that exhibits versatile and highly flexible connectivity patterns with disparate brain networks [75], we believe that characterizing its dynamic connectivity patterns (i.e, modular architectures of evolving networks analyses, also called time-varying changes in functional connectivity) would be a more ecologically valid and informative method for understanding the timing of the balance between the implicated networks.

## Figures and Tables

**Table 1:** Main characteristics of included studies comparing future abstainers and relapsers.

		Authors and N°	Population; % of Abstainers	Treatment	Duration of ttt; End Point; Abstinence Criteria	Scans: All Had at Least One MRI Before the Treatment	Interpretation of the Authors
A N A T O M I C A L		Froeliger-2010 **1**	N = 18 (16w; 4 m) 39.06 yo (9.37) 10R/8A 44.44%	Single arm study NRT (RNC + Patch)	Treatment: 10 weeks End point: 4 weeks point prevalence abstinence Criteria: - Self-reported abstinence in the 21 days leading up to the 4 weeks clinic visit - CO ≤ 8 ppm	Whole Brain 1.5 T GE NVi SIGNA scanner	Results: Relapse was associated with lower GMV in the left putamen and right occipital lobe and greater GMV in bilateral hippocampus and right cuneus. Maintaining smoking abstinence is associated with higher prequit brain volume in regions that subserve habit learning and visual processing, and lower brain volume in regions that subserve long-term memory processes and visual information processing. Covariates: years smoked and cigarettes per day as covariates, Total Intracranial Volume TIV and sex.
		Froeliger-2017 **2**	N = 81 40R (21 w;19 m) 41A (24 w;17 m) 50.62%	Randomized control trial Before the quit date: Two-arms 30 days smoking cessation program Gp1: continue smoking Gp2: low nicotine cigarettes + NRT (patch) After the quit date: NRT (patch) + PCS both groups	Treatment: 10 weeks End point: 10 weeks Criteria: - Daily diaries of cigarette use - CO < 8 ppm - relapse was defined as 7 days consecutive of smoking at least 1 cig/day	Whole Brain 3T scanners (Signa Excite HD and MR750; GE Healthcare). Contingency management has been used during the post-scan behavioral task.	Results: Relapsers had lower GMV in right IFG. Together, these findings suggest that reduced prefrontal gray matter volume may reduce inhibitory control over behavioral response to conditioned drug-cues. This finding is consistent with the extant literature implicating drug addiction-related neuroplasticity in fronto-striatal circuitry, which mediates cue-induced relapse as being associated with frontally mediated behavior inhibition. Covariates: FTND (nuisance covariate)
		Wang–2019a **3**	N = 74 All men 46R/28A 37.83%	Single arm study Pharm (Varenicline) +PCS	Treatment: 12 weeks End point: last 4 weeks Criteria: - weekly self-reports of smoking behavior - CO ≤ 6 ppm	ROI: Insula (anterior and posterior) 3.0 T GE Signa MR scanner GMV + SCN	Results: No association Smoking cessation outcomes showed no correlations with the gray matter volume and seed-based structural covariance network of insular subregions prior to smoking cessation. Covariates: Not reported
		Qian–2019 **4**	N = 73 All men 44R/29A 39.73%	Single arm study Pharm (Varenicline) + PCS	Treatment: 12 weeks End point: last 4 weeks Criteria: Continuously abstinent for the last 4 weeks of treatment - Weekly self-reports of smoking behavior - CO ≤ 6 ppm	Whole brain + ROI (OFC, dorsal striatum, postcentral gyrus, thalamus) 3.0 T GE Signa MR scanner	Results: Relapsers had lower GMV in the right post-central gyrus, right dorsal striatum, and left OFC. Structural integrity of OFC is important for quitting smoking. Smaller dorsal striatum grey matter volume may be associated with a disturbance of DA functions, and it may contribute to the neurobiology of nicotine abstinence symptomatology. Covariates: Age and education years.
		Wang–2020 **5**	N = 74 All men 47R/27A 36.49%	Single arm study Pharm (Varenicline) + PCS	Treatment: 12 weeks End point: last 4 weeks Criteria: Continuously abstinent for the last 4 weeks of treatment - Weekly self-reports of smoking behavior - CO ≤ 6 ppm	ROI: Thalamus 3.0 T GE Signa MR scanner	Results: Relapsers had greater left thalamic GMV. Pre-existing abnormalities in the thalamus may potentially predispose individuals to the initiation of smoking and the development of nicotine dependence, such as the genetic factor. Covariates: Not reported.
		Huang–2017 **6**	N = 66 All men 38R/28A 42.42%	Single arm study Pharm (Varenicline) + PCS	Treatment: 12 weeks End point: last 4 weeks Criteria: Continuously abstinent for the last 4 weeks of treatment - Weekly self-reports of smoking behavior - CO ≤ 6 ppm	Whole Brain 3.0 T GE Signa MR scanner	Results: Relapsers had higher fractional anisotropy in the right cerebellum and in post-central gyrus. Considering the general function of these two structures and the related evidence, we suggested that the higher FA may indicate the formation of habitual/automatic smoking behaviors that promote smoking relapse. Covariates: Age and education years.
		Wang–2021a **7**	N = 58 All men 38R/20A 34.48%	Single arm study Pharm (Varenicline) + PCS	Treatment: 12 weeks End point: last 4 weeks Criteria: Continuously abstinent for the last 4 weeks of treatment - Weekly self-reports of smoking behavior - CO ≤ 6ppm	ROI: Insular seed regions and OFC and NAc targets 3.0 T GE Signa MR scanner	Results: No association Cessation likelihood may be more susceptible to insula-related functional connectivity than insula-related structural connectivity. Covariates: Not reported
R E S T I N G - S T A T E		Sweitzer–2016 (b) **8**	N = 37 23R (13 w;10 m) 14A (6 w; 8 m) 37.84%	Single arm study PCS (Contingency management)	Treatment: 3 weeks Endpoint: 18 days of abstinence Criteria: - Any smoking after achieving an initial 24 h abstinence (ie, two consecutive abstinent samples), - Self-report - CO ≤ 8 ppm or a 50% reduction from baseline	2 randomized MRI (condition: satiety and 24 h of abstinence) Whole brain: connectivity with ventral and dorsal striatum seed regions	Modulation of striatal connectivity with the cingulo insula network during early withdrawal may be associated with smoking cessation outcomes. Covariates: Age, sex, cigarettes smoked per day, and session order (i.e., abstinent or satiated condition first).
		Addicott–2015 **9**	N = 85 41R (23 w;18 m) 44A (23 w;22 m) 51.76%	Randomized control trial Before the quit date: Two-arms 30 days smoking cessation program Gp1: continue smoking Gp2: low nicotine cigarettes + NRT (patch) After the quit date: NRT (patch) + PCS both groups	Treatment: 10 weeks Endpoint: 10 weeks Criteria: - relapse = 7 days consecutive of smoking cigarettes (at least one per/day) following the quit day or lost to follow-up (N = 14 *) - Daily diaries of cigarette and NRT use + expired CO on four occasions spaced across the 10 weeks of post-quit date treatment	1 MRI in satiated state Whole brain: connectivity with bilateral posterior, ventroanterior and dorsoanterior insula seed regions.	Relapse vulnerability is associated with weaker connectivity between the posterior insula and primary sensorimotor cortices. Perhaps greater connectivity in this network improves the ability to inhibit a motor response to cigarette cravings when those cravings conflict with a goal to remain abstinent. Covariates: Treatment group and FTND scores.
		Wilcox–2017 **10**	N = 144 (53 w;91 m) N = 82 varenicline treatment, N = 62 placebo 120R/24A 16.67%	Randomized control trial Pharm (Varenicline) Gp1: Varenicline + PCS Gp2: Placebo + PCS	Treatment: 12 weeks End point: 12 weeks Criteria: - Number of cigarettes in previous 30 days	1 MRI in abstinence state (N = 8 were not) ROI network: - 6 networks utilized Dorsolateral PFC, dorsal ACC, rostral ACC, Insula, Caudate, Putamen	No main effect of treatment group. Covariates: Treatment group and baseline smoking.
		Qian–2019 **4**	N = 73 44R/29A 39.73%	Single arm study Pharm (Varenicline)+ PCS	Treatment: 12 weeks End point: last 4 weeks Criteria: Continuously abstinent for the last 4 weeks of treatment - Weekly self-reports of smoking behavior - CO ≤ 6 ppm	1 MRI in satiated state Whole brain connectivity with OFC, dorsal striatum, postcentral gyrus and thalamic seed regions	Decreased thalamus-cerebellar FC may hinder the communication between the frontal lobe and the cerebellum, invalidating the top-down regulations. Therefore, the relapsers may experience difficulties in utilizing cognitive abilities to reverse habitual behaviors. Covariates: Age and education years.
		Wang–2020 **5**	N = 74 All men 47R/27A(#) 36.49%	Single arm study Pharm (Varenicline) + PCS	Treatment: 12 weeks End point: last 4 weeks Criteria: Continuously abstinent for the last 4 weeks of treatment - Weekly self-reports of smoking behavior - CO ≤ 6 ppm	1 MRI in satiated state Whole brain: connectivity with thalamic seed regions	Relapsers showed lower left thalamo-precuneus functional connectivity. Thalamo-precuneus functional connectivity degradation may be associated with both the maintenance of smoking behavior and smoking relapse. Covariates: White matter signal and corticospinal fluid signal in addition to head motion (nuisance covariates).
		Wang–2019b **11**	N = 30 All men 14R/16A 53.33%	Single arm study Pharm (Varenicline) + PCS	Treatment: 12 weeks End point: last 4 weeks Criteria: Continuously abstinent for the last 4 weeks of treatment - Weekly self-reports of smoking behavior - CO ≤ 6 ppm	2 MRI scanning sessions in satiated state: - at baseline - after treatment Whole brain: connectivity with insula seed regions.	Altered interregional functional connectivity but not regional activity of insular subregions is associated with smoking cessation outcome. Increased FC network of the anterior insula could help resist relapse to improve smoking cessation likelihood. Covariates: White matter signal and corticospinal fluid signal in addition to head motion (nuisance covariates). Age was added in the fALFF analysis.
		Wang–2021b **12**	N = 30 All men 14R/16A 53.33%	Single arm study Pharm (Varenicline) + PCS	Treatment: 12 weeks End point: last 4 weeks Criteria: Continuously abstinent for the last 4 weeks of treatment - Weekly self-reports of smoking behavior - CO ≤ 6 ppm	2 MRI scanning sessions in satiated state: - at baseline - after treatment Whole brain: connectivity with striatum (NAc, caudate, putamen) seed regions.	Lower NAc-based functional connectivity with the frontoinsular areas may reflect both a lower awareness of subjective urge to smoke and a lower ability of cognitive control for maintaining abstinence. Covariates: White matter signal and corticospinal fluid signal in addition to head motion (nuisance covariates); Age.
		Wang–2017 **13**	N = 55 All men 32R/23A 41.82%	Single arm study Pharm (Varenicline) + PCS	Treatment: 12 weeks End point: 12 weeks Criteria: Continuously abstinent for 12 weeks - weekly self-reports of smoking behavior - CO ≤ 6 ppm	1 MRI in satiated state Whole brain: Reho	Relapsers had decreased ReHo in the bilateral PCC and increased ReHo in the left STG, suggesting that regional brain function variables may be promising predictors of smoking relapse. Covariates: Years of education, years smoked, cigarettes smoked per day and FTND score.
		Shen–2017 **14**	N = 57 36R/21A 36.84%	Single arm study Pharm (Varenicline) + PCS	Treatment: 12weeks End point: last 4 weeks Criteria: Continuously abstinent for the last 4 weeks of treatment - Weekly self-reports of smoking behavior - CO ≤ 6 ppm	1 MRI in satiated state Whole brain: Eigenvector centrality (EC) mapping.	These findings suggest that the dlPFC, MTG, and cerebellum may be important substrates of smoking relapse vulnerability. The data also suggest that relapse-vulnerable smokers can be identified before quit attempts, which could enable personalized treatment and improve smoking cessation outcomes. Covariates: Years smoked, cigarettes per day.
T A S K - B A S E D A C T I V A T I O N	I N H I B I T I O N	Froeliger–2017 (Study 1) **2**	N = 81 40R (21 w;19 m) 41A (24 w;17 m) 50.62%	Randomized control trial Before the quit date: Two-arms 30 days smoking cessation program Gp1: continue smoking Gp2: low nicotine cigarettes + NRT (patch) After the quit date: NRT (patch) + PCS both groups	Treatment: 10 weeks End point: 10 weeks Criteria: - Daily diaries of cigarette use - CO < 8 ppm - relapse was defined as 7 days consecutive of smoking at least 1 cig/day	1 MRI in satiated state ROI: Right IFG, bilateral thalamus, subthalamic nucleus, presupplementary motor area and left primary motor cortex Contingency management has been used during the post-scan behavioral task.	Individual differences in corticothalamic circuitry function have important implications for smoking cessation and relapse vulnerability. Covariates: Baseline FTND.
		Gilman–2018 **15**	N = 22 12R (4 w;8 m) 10A (2 w;8 m) 45.45%	Randomized control trial Pharm (EVP-6124 or placebo) + NRT (patch or placebo)+ PCS for all groups Gp1: EVP-6124 + NRT Gp2: EVP-placebo + NRT Gp3: EVP-6124 + NRT-placebo Gp4: EVP-placebo + NRT-placebo	Treatment: 12 weeks End point: 2 weeks Criteria: - Self-report of 2 weeks abstinence - CO < 10 ppm - Cotinine < 50 ng/mL.	1 MRI in satiated state ROI: bilateral ant insula, right IFG	Results from the current study suggest that while brain activation during inhibition to smoking cues does not significantly differ from inhibition to neutral cues, decreased activation in the anterior insula to inhibition of smoking cues may be associated with relapse among smokers attempting to remain abstinent. Covariates: FTND.
	D R U G C U E S	McClernon–2007 **16**	N = 16 (14 w;2 m) 12R/4A 25.00%	Single arm study NRT (RNC + Patch) + PCS	Treatment: 8 to 9 weeks End point: 1 month Criteria: - Self-reports of no cigarette smoking since the quit day - CO < 9 ppm	3 MRI (at baseline; quit date; 2-4weeks of abstinence) ROI: ventral and dorsal ACG, SFG, MFG, IFG, NAc, thalamus, caudate, putamen, amygdala, hippocampus, insula, ventral striatum (NAc+central caudate and putamen)	An extinction-based smoking cessation treatment could alter brain responses to smoking cues and these changes may be associated with treatment outcome. Covariates: Not reported.
		Janes–2010 **17**	N = 21 All women 9R/12A 57.14%	Single arm study NRT: (Patch+ nicotine gum (lozenge)) + PCS	Treatment: 8 weeks End point: 8 weeks Criteria: - 7 days or more consecutive or more than once/week for 2 or more consecutive weeks - Self-reported abstinence - CO < 9 ppm	1 MRI Whole brain	Insula and amygdala activation might imply that smoking-related images are more emotionally salient and may induce interoceptive awareness to a greater extent than neutral images in smokers vulnerable to relapse. (In addition…) vulnerable subjects may be more likely to prepare for, or initiate, motor responses geared toward reducing interoceptive sensations related to craving. Covariates: Not reported
		Owens–2018 **18**	N = 32 23R/9A for 49 days of treatment 28.13% **	Single arm study NRT (Patch) + PCS	Treatment: 9 weeks End point: 49 days Criteria: - Self-reported timeline follow back calendar - CO < 10 ppm At each session Lost of follow-up are in relapser group	1 MRI (2 h of abstinence) ROI: R&L ventral striatum, caudal and rostral ACC, left amygdala	Greater in ventral striatum, amygdala and ACC was associated with less difficulty quitting, suggesting their activity is an indicator of less severe risk for lapse. Clinically, this implies that for smokers who have transitioned from incentive learning and more deliberative cognitive processing to having habits learning and automatized drug seeking, smoking cessation is more difficult. Covariates: Self-reported craving prior and during the MRI session.
		Hartwell–2013 **19**	N = 21 (12 w;9 m) 11R/10A 47.62%	Single arm study Pharm (Varenicline) + PCS	Treatment: 5 weeks End point: 5 weeks Criteria: - 7 days point prevalence abstinence - CO ≤ 3 ppm - Cotinine < 200 ng/mL	2 MRI: - 1 at baseline (resist and crave during scan) - 1 month after quit date Whole brain ***	Successful smoking cessation with varenicline is associated with increased activation, prior to a quit attempt, in brain areas related to attentiveness and memory while resisting the urge to smoke. Covariates: Not reported.
		Janes–2017 **20**	N = 23 (7 w;16 m) 13R/10A 43.48%	Randomized control trial Pharm (EVP-6124 or placebo) + NRT (patch or placebo) + PCS for all groups Gp1: EVP-6124 + NRT Gp2: EVP-placebo + NRT Gp3: EVP-6124 + NRT-placebo Gp4: EVP-placebo + NRT-placebo	Treatment: 12 weeks End point: 2 weeks Criteria: - Self-report of 2 weeks abstinence - CO < 10 ppm, - Cotinine < 50 ng/mL.	1 MRI in satiated state ROI: Bilate insula, striatum (nucleus accumbens, putamen, caudate), and thalamus.	The current work supports prior results and builds on the concept that the insula and dorsal striatum work in concert to maintain nicotine dependence. Covariates: Not reported
		Allenby–2020 **21**	N = 75 (35 w;40 m) 52R/23A 30.67%	Single arm study PCS	Treatment: 1 weeks End point: 1 weeks Criteria: - Self-report abstinence - Urine cotinine < 100 ng/mL - CO ≤ 5 ppm	2 MRI (satiety and 24 h of abstinence) Whole brain	This study provides the first evidence that changes in smoking cue reactivity in the ACC during acute abstinence predict smoking relapse, thereby improving our understanding of the neurobiology of smoking cessation. Covariates: Abstinence-induced changes in craving and withdrawal, sex, age, baseline smoking (cigarettes per day and CO at intake).
	H E A L T H Y M E S S A G E	Chua–2011 **22**	N = 91 (44 w,47 m) 45A/42R 4 lost 51.72%	Single arm study NRT (Patch) + PCS	Treatment: 10 weeks End point: 4 months after the intervention Criteria: - Self-reported 7 days point prevalence abstinence	2 MRI (two tasks) Whole brain **+** ROI: (dorsomedial PFC, precuneus/PCC, angular gyrus)	The dmPFC region has been associated with the evaluative and decision-making aspect of self-related processing which could underlie the efficacy of tailored message interventions. Covariates: Number of cigarettes per day at baseline.
		Owens–2017 **23**	N = 48 (17 w;31 m) 24R/24A 50.00%	Single arm study NRT (Patch) + PCS	Treatment: 9 weeks End point: 30 days Criteria: - Self reported - CO ≤ 10 ppm	1 MRI (2 h of abstinence) ROI: vmPFC, Right and Left amygdala	Neurocognitive processes in the vmPFC may be critical to understanding how Graphic Warning Labels on cigarette packaging induce behavior changes and may be useful as a predictor of smoking cessation treatment prognosis. Covariates: FTND
	R E W A R D	Sweitzer–2016 (a) **24**	N = 36 (19 w;17 m) 23R/13A 36.11%	Single arm study PCS Contingency management during treatment	Treatment: 3 weeks End point: 18 days of abstinence Criteria: - Self-reported abstinence - CO < 8 ppm or a 50% reduction from baseline	2 randomized MRI (condition: satiety and 24 h of abstinence) ROI: Right and Left striatum (head of the caudate)	Results support the importance of blunted reward sensitivity as a risk factor for relapse and highlight the moderating effect of abstinence state. Early abstinence may be a particular period of vulnerability. Covariates: Age, sex, abstinence-induced craving, and baseline cigarette per day
		Grosskopf–2020 **25**	N = 44 (21 w;23 m) 19R/25A 56.82%	Single arm study PCS	Treatment: length not reported End point: 30 days Criteria: - Smoking less than 1 day over a 30-day period - CO verified	2 MRI: -1 at baseline in satiated state - 2 to 5 weeks after quit date Whole brain:	Tobacco abstinence did not affect discounting behavior as well as related fMRI brain activity in smokers. Covariates: FNTD and discounting behavior.
	F U N C T I O N A L C O N N E C T I V I T Y	Janes–2010 **17**	N = 21 All women9R/12A 57.14%	Single arm study NRT: (Patch+ nicotine gum (lozenge)) + PCS	Treatment: 8 weeks End point: 8 weeks Criteria: - 7 days or more consecutive or more than once/week for 2 or more consecutive weeks - Self-reported abstinence - CO < 9 ppm	1 MRI ROI: ICA defined: Bilateral insula et ACC	Relapsers may have decreased top-down control of emotion regulation. This could result in increased interoceptive awareness of smoking-related cues, leading to enhanced smoking cue reactivity, interference effects, and relapse vulnerability. Covariates: Not reported.
		Froeliger–2017 **2**	N = 81 40R (21 w;19 m) 41A (24w;17m) 50.62%	Randomized control trial Before the quit date: Two-arms 30 days smoking cessation program Gp1: continue smoking Gp2: low nicotine cigarettes + NRT (patch) After the quit date: NRT (patch) + PCS both groups	Treatment: 10 weeks End point: 10 weeks Criteria: - Daily diaries of cigarette use - CO < 8 ppm - Relapse was defined as 7 days consecutive of smoking at least 1 cigarette/day	1 MRI ROI: Thalamus Inferior frontal gyrus Contingency management has been used during the post-scan behavioral task.	Baseline differences in corticothalamic circuitry function were associated with mediated IC and smoking relapse vulnerability. These findings warrant further examination of interventions for augmenting corticothalamic neurotransmission and enhancing inhibitory control during the course of tobacco use disorder treatment. Covariates: FTND (nuisance covariate).

At baseline = before the treatment; ppm: parts per million; NRT: Nicotine replacement therapy; PCS: Psychological counselling session; RNC: Reduced nicotine cigarettes; Pharm.: Pharmacotherapy; (#) Inconsistent reporting of the number of relapsers: 47 are announced in the Statistical analyses section while 54 are mentioned in the Thalamus based rsFC Result Section; * = No information is provided on the proportion of Gp1 or Gp2 among these 14 participants lost to follow-up; ** = The abstinence rate did not take into account the fact that 14/46 participants did not initiate a quit attempt so the adjusted abstinence prevalence is 19.57%; *** = 2 participants were excluded from the MRI analyses. As no information on whether these participants were abstainers or relapsers, we do not know how many abstainers versus relapsers were included in the final MRI analyses. Gp: Group; w: women; m: men; CO: Carbone monoxide; ROI: Region of Interest; fALFF: Fractional amplitude of low frequency fluctuation; dl: dorsolateral; vm: ventro medial; ACC: Anterior Cingulate Cortex; ACG: Anterior Cingulate Gyrus; IFG: Inferior Frontal Gyrus; MTG: Middle Temporal Gyrus; MFG: Middle Frontal Gyrus; NAc: nucleus Accumbens; OFC: Orbito-Frontal Cortex; PCC: Posterior Cingulate Cortex; PFC: Prefrontal Cortex; SFG: Superior Frontal Gyrus; FTND: Fagerstrom Test for Nicotine Dependance.

## Data Availability

Not applicable.

## Supplementary Materials

The following are available online at https://www.mdpi.com/article/10.3390/biology11010035/s1, Figure S1: Study flow diagram.

## Abbreviations

ACC	anterior cingular cortex
DMN	Default Mode Network
FTND	Fagerstrom Test for Nicotine Dependence
GMV	grey matter volume
IC	inhibitory control
IFG	inferior frontal gyrus
MFG	middle frontal gyrus
MRI	Magnetic Resonance Imaging
MTG	middle temporal gyrus
NRT	nicotine replacement therapies
PCC	posterior cingulate cortex
PCS	psychological counselling sessions
PFC	prefrontal cortex
ReHo	regional homogeneity
ROIs	regions of interests
RS	resting-state
SFG	superior frontal gyrus
STG	superior temporal gyrus
STG	superior temporal gyrus
tbFC	task-based functional connectivity
VBM	voxel-based morphometry
WB	whole brain

## References

[1] Reitsma M.B., Kendrick P.J., Ababneh E., Abbafati C., Abbasi-Kangevari M., Abdoli A., Abedi A., Abhilash E.S., Abila D.B., Aboyans V. (2021). Spatial, Temporal, and Demographic Patterns in Prevalence of Smoking Tobacco Use and Attributable Disease Burden in 204 Countries and Territories, 1990–2019: A Systematic Analysis from the Global Burden of Disease Study 2019. Lancet.

[2] Ribassin-Majed L., Hill C. (2015). Trends in Tobacco-Attributable Mortality in France. Eur. J. Public Health.

[3] Hughes J.R., Keely J., Naud S. (2004). Shape of the Relapse Curve and Long-Term Abstinence among Untreated Smokers. Addiction.

[4] Livingstone-Banks J., Norris E., Hartmann-Boyce J., West R., Jarvis M., Hajek P. (2019). Relapse Prevention Interventions for Smoking Cessation. Cochrane Database Syst. Rev..

[5] Piazza P.V., Deroche-Gamonet V. (2013). A Multistep General Theory of Transition to Addiction. Psychopharmacology.

[6] Mathew A.R., Hogarth L., Leventhal A.M., Cook J.W., Hitsman B. (2017). Cigarette Smoking and Depression Comorbidity: Systematic Review & Proposed Theoretical Model. Addiction.

[7] Fatseas M., Serre F., Alexandre J.-M., Debrabant R., Auriacombe M., Swendsen J. (2015). Craving and Substance Use among Patients with Alcohol, Tobacco, Cannabis or Heroin Addiction: A Comparison of Substance- and Person-Specific Cues: Cues, Craving and Substance Use. Addiction.

[8] Cahill K., Stevens S., Perera R., Lancaster T. (2013). Pharmacological Interventions for Smoking Cessation: An Overview and Network Meta-Analysis. Cochrane Database Syst. Rev..

[9] Garcia-Rivas V., Deroche-Gamonet V. (2019). Not All Smokers Appear to Seek Nicotine for the Same Reasons: Implications for Preclinical Research in Nicotine Dependence. Addict. Biol..

[10] Wang C., Xu X., Qian W., Shen Z., Zhang M. (2015). Altered Human Brain Anatomy in Chronic Smokers: A Review of Magnetic Resonance Imaging Studies. Neurol. Sci..

[11] Zhou S., Xiao D., Peng P., Wang S.-K., Liu Z., Qin H.-Y., Li S.-S., Wang C. (2017). Effect of Smoking on Resting-State Functional Connectivity in Smokers: An FMRI Study: Effects of Smoking on Brain Function. Respirology.

[12] Heatherton T.F., Kozlowski L.T., Frecker R.C., Fagerstrom K.-O. (1991). The Fagerstrom Test for Nicotine Dependence: A Revision of the Fagerstrom Tolerance Questionnaire. Addiction.

[13] National Research Council (US) Committee on A Framework for Developing a New Taxonomy of Disease (2011). Toward Precision Medicine: Building a Knowledge Network for Biomedical Research and a New Taxonomy of Disease.

[14] Naqvi N.H., Rudrauf D., Damasio H., Bechara A. (2007). Damage to the Insula Disrupts Addiction to Cigarette Smoking. Science.

[15] Froeliger B., McConnell P.A., Bell S., Sweitzer M., Kozink R.V., Eichberg C., Hallyburton M., Kaiser N., Gray K.M., McClernon F.J. (2017). Association Between Baseline Corticothalamic-Mediated Inhibitory Control and Smoking Relapse Vulnerability. JAMA Psychiatry.

[16] Froeliger B., Kozink R.V., Rose J.E., Behm F.M., Salley A.N., McClernon F.J. (2010). Hippocampal and Striatal Gray Matter Volume Are Associated with a Smoking Cessation Treatment Outcome: Results of an Exploratory Voxel-Based Morphometric Analysis. Psychopharmacology.

[17] Wang C., Huang P., Shen Z., Qian W., Li K., Luo X., Zeng Q., Guo T., Yu H., Yang Y. (2019). Gray Matter Volumes of Insular Subregions Are Not Correlated with Smoking Cessation Outcomes but Negatively Correlated with Nicotine Dependence Severity in Chronic Smokers. Neurosci. Lett..

[18] Qian W., Huang P., Shen Z., Wang C., Yang Y., Zhang M. (2019). Brain Gray Matter Volume and Functional Connectivity Are Associated With Smoking Cessation Outcomes. Front. Hum. Neurosci..

[19] Wang C., Wang S., Shen Z., Qian W., Jiaerken Y., Luo X., Li K., Zeng Q., Gu Q., Yang Y. (2020). Increased Thalamic Volume and Decreased Thalamo-Precuneus Functional Connectivity Are Associated with Smoking Relapse. NeuroImage Clin..

[20] Good C.D., Johnsrude I.S., Ashburner J., Henson R.N.A., Friston K.J., Frackowiak R.S.J. (2001). A Voxel-Based Morphometric Study of Ageing in 465 Normal Adult Human Brains. NeuroImage.

[21] Huang P., Shen Z., Wang C., Qian W., Zhang H., Yang Y., Zhang M. (2017). Altered White Matter Integrity in Smokers Is Associated with Smoking Cessation Outcomes. Front. Hum. Neurosci..

[22] Wang C., Wang S., Huang P., Shen Z., Qian W., Luo X., Li K., Zeng Q., Gu Q., Yu H. (2021). Abnormal White Matter Tracts of Insula in Smokers. Brain Imaging Behav..

[23] Biswal B.B. (2012). Resting State FMRI: A Personal History. NeuroImage.

[24] Buckner R.L., Krienen F.M. (2013). The Evolution of Distributed Association Networks in the Human Brain. Trends Cogn. Sci..

[25] Sweitzer M., Geier C.F., Denlinger R., Forbes E.E., Raiff B.R., Dallery J., McClernon F.J., Donny E.C. (2016). Blunted Striatal Response to Monetary Reward Anticipation during Smoking Abstinence Predicts Lapse during a Contingency-Managed Quit Attempt. Psychopharmacology.

[26] Addicott M.A., Sweitzer M.M., Froeliger B., Rose J.E., McClernon F.J. (2015). Increased Functional Connectivity in an Insula-Based Network Is Associated with Improved Smoking Cessation Outcomes. Neuropsychopharmacology.

[27] Wilcox C.E., Calhoun V.D., Rachakonda S., Claus E.D., Littlewood R.A., Mickey J., Arenella P.B., Hutchison K.E. (2017). Functional Network Connectivity Predicts Treatment Outcome during Treatment of Nicotine Use Disorder. Psychiatry Res. Neuroimaging.

[28] Wang C., Shen Z., Huang P., Qian W., Zhou C., Li K., Zeng Q., Luo X., Gu Q., Yu H. (2019). Increased Interregional Functional Connectivity of Anterior Insula Is Associated with Improved Smoking Cessation Outcome. Brain Imaging Behav..

[29] Wang C., Huang P., Shen Z., Qian W., Wang S., Jiaerken Y., Luo X., Li K., Zeng Q., Zhou C. (2021). Increased Striatal Functional Connectivity Is Associated with Improved Smoking Cessation Outcomes: A Preliminary Study. Addict. Biol..

[30] Zang Y., Jiang T., Lu Y., He Y., Tian L. (2004). Regional Homogeneity Approach to FMRI Data Analysis. NeuroImage.

[31] Wang C., Shen Z., Huang P., Qian W., Yu X., Sun J., Yu H., Yang Y., Zhang M. (2017). Altered Spontaneous Activity of Posterior Cingulate Cortex and Superior Temporal Gyrus Are Associated with a Smoking Cessation Treatment Outcome Using Varenicline Revealed by Regional Homogeneity. Brain Imaging Behav..

[32] Lohmann G., Margulies D.S., Horstmann A., Pleger B., Lepsien J., Goldhahn D., Schloegl H., Stumvoll M., Villringer A., Turner R. (2010). Eigenvector Centrality Mapping for Analyzing Connectivity Patterns in FMRI Data of the Human Brain. PLoS ONE.

[33] Shen Z., Huang P., Wang C., Qian W., Yang Y., Zhang M. (2017). Increased Network Centrality as Markers of Relapse Risk in Nicotine-Dependent Individuals Treated with Varenicline. Prog. Neuro-Psychopharmacol. Biol. Psychiatry.

[34] Gilman J.M., Radoman M., Schuster R.M., Pachas G., Azzouz N., Fava M., Evins A.E. (2018). Anterior Insula Activation during Inhibition to Smoking Cues Is Associated with Ability to Maintain Tobacco Abstinence. Addict. Behav. Rep..

[35] Courtney K.E., Schacht J.P., Hutchison K., Roche D.J.O., Ray L.A. (2016). Neural Substrates of Cue Reactivity: Association with Treatment Outcomes and Relapse: Cue Reactivity and Outcomes. Addict. Biol..

[36] Hartwell K.J., LeMatty T., McRae-Clark A.L., Gray K.M., George M.S., Brady K.T. (2013). Resisting the Urge to Smoke and Craving during a Smoking Quit Attempt on Varenicline: Results from a Pilot FMRI Study. Am. J. Drug Alcohol Abus..

[37] Janes A.C., Pizzagalli D.A., Richardt S., de Frederick B.B., Chuzi S., Pachas G., Culhane M.A., Holmes A.J., Fava M., Evins A.E. (2010). Brain Reactivity to Smoking Cues Prior to Smoking Cessation Predicts Ability to Maintain Tobacco Abstinence. Biol. Psychiatry.

[38] Allenby C., Falcone M., Wileyto E.P., Cao W., Bernardo L., Ashare R.L., Janes A., Loughead J., Lerman C. (2020). Neural Cue Reactivity during Acute Abstinence Predicts Short-term Smoking Relapse. Addict. Biol..

[39] McClernon F.J., Hiott F.B., Liu J., Salley A.N., Behm F.M., Rose J.E. (2007). Selectively Reduced Responses to Smoking Cues in Amygdala Following Extinction-Based Smoking Cessation: Results of a Preliminary Functional Magnetic Resonance Imaging Study. Addict. Biol..

[40] Owens M.M., MacKillop J., Gray J.C., Beach S.R.H., Stein M.D., Niaura R.S., Sweet L.H. (2018). Neural Correlates of Tobacco Cue Reactivity Predict Duration to Lapse and Continuous Abstinence in Smoking Cessation Treatment: Neural Correlates of Tobacco Cue Reactivity. Addict. Biol..

[41] Janes A.C., Gilman J.M., Radoman M., Pachas G., Fava M., Evins A.E. (2017). Revisiting the Role of the Insula and Smoking Cue-Reactivity in Relapse: A Replication and Extension of Neuroimaging Findings. Drug Alcohol Depend..

[42] Chua H.F., Ho S.S., Jasinska A.J., Polk T.A., Welsh R.C., Liberzon I., Strecher V.J. (2011). Self-Related Neural Response to Tailored Smoking-Cessation Messages Predicts Quitting. Nat. Neurosci..

[43] Owens M.M., MacKillop J., Gray J.C., Hawkshead B.E., Murphy C.M., Sweet L.H. (2017). Neural Correlates of Graphic Cigarette Warning Labels Predict Smoking Cessation Relapse. Psychiatry Res. Neuroimaging.

[44] Sweitzer M.M., Geier C.F., Addicott M.A., Denlinger R., Raiff B.R., Dallery J., McClernon F.J., Donny E.C. (2016). Smoking Abstinence-Induced Changes in Resting State Functional Connectivity with Ventral Striatum Predict Lapse During a Quit Attempt. Neuropsychopharmacology.

[45] Grosskopf C.M., Kroemer N.B., Pooseh S., Böhme F., Smolka M.N. (2021). Temporal Discounting and Smoking Cessation: Choice Consistency Predicts Nicotine Abstinence in Treatment-Seeking Smokers. Psychopharmacology.

[46] Friston K.J. (2011). Functional and Effective Connectivity: A Review. Brain Connect..

[47] Naqvi N.H., Bechara A., Heather N., Segal G. (2016). The Role of the Insula in Goal-Directed Drug Seeking and Choice in Addiction. Addiction and Choice.

[48] Abdolahi A., Williams G.C., Benesch C.G., Wang H.Z., Spitzer E.M., Scott B.E., Block R.C., van Wijngaarden E. (2015). Smoking Cessation Behaviors Three Months Following Acute Insular Damage from Stroke. Addict. Behav..

[49] Suñer-Soler R., Grau A., Gras M.E., Font-Mayolas S., Silva Y., Dávalos A., Cruz V., Rodrigo J., Serena J. (2012). Smoking Cessation 1 Year Poststroke and Damage to the Insular Cortex. Stroke.

[50] Abdolahi A., Williams G.C., Benesch C.G., Wang H.Z., Spitzer E.M., Scott B.E., Block R.C., van Wijngaarden E. (2015). Damage to the Insula Leads to Decreased Nicotine Withdrawal during Abstinence: Insular Damage and Withdrawal during Abstinence. Addiction.

[51] Abdolahi A., Williams G.C., Benesch C.G., Wang H.Z., Spitzer E.M., Scott B.E., Block R.C., van Wijngaarden E. (2017). Immediate and Sustained Decrease in Smoking Urges After Acute Insular Cortex Damage. Nicotine Tob. Res..

[52] Menon V., Uddin L.Q. (2010). Saliency, Switching, Attention and Control: A Network Model of Insula Function. Brain Struct Funct.

[53] Hariri A.R., Brown S.M., Williamson D.E., Flory J.D., de Wit H., Manuck S.B. (2006). Preference for Immediate over Delayed Rewards is Associated with Magnitude of Ventral Striatal Activity. J. Neurosci..

[54] Luijten M., Schellekens A.F., Kühn S., Machielse M.W.J., Sescousse G. (2017). Disruption of Reward Processing in Addiction: An Image-Based Meta-Analysis of Functional Magnetic Resonance Imaging Studies. JAMA Psychiatry.

[55] David S.P., Munafò M.R., Johansen-Berg H., Smith S.M., Rogers R.D., Matthews P.M., Walton R.T. (2005). Ventral Striatum/Nucleus Accumbens Activation to Smoking-Related Pictorial Cues in Smokers and Nonsmokers: A Functional Magnetic Resonance Imaging Study. Biol. Psychiatry.

[56] Franklin T.R., Wang Z., Wang J., Sciortino N., Harper D., Li Y., Ehrman R., Kampman K., O’Brien C.P., Detre J.A. (2007). Limbic Activation to Cigarette Smoking Cues Independent of Nicotine Withdrawal: A Perfusion FMRI Study. Neuropsychopharmacol.

[57] Wang Z., Faith M., Patterson F., Tang K., Kerrin K., Wileyto E.P., Detre J.A., Lerman C. (2007). Neural Substrates of Abstinence-Induced Cigarette Cravings in Chronic Smokers. J. Neurosci..

[58] Jing C., Jing C., Zheng L., Hong G., Zheng J., Yu L., Song N., Zhang T., Ma Q., Fang J. (2021). Disruption of Cigarette Smoking Addiction After Dorsal Striatum Damage. Front. Behav. Neurosci..

[59] Gaznick N., Tranel D., McNutt A., Bechara A. (2014). Basal Ganglia plus Insula Damage Yields Stronger Disruption of Smoking Addiction than Basal Ganglia Damage Alone. Nicotine Tob. Res..

[60] Noël X., Brevers D., Bechara A. (2013). A Triadic Neurocognitive Approach to Addiction for Clinical Interventions. Front. Psychiatry.

[61] Piasecki T.M. (2006). Relapse to Smoking. Clin. Psychol. Rev..

[62] Alboksmaty A., Agaku I.T., Odani S., Filippidis F.T. (2019). Prevalence and Determinants of Cigarette Smoking Relapse among US Adult Smokers: A Longitudinal Study. BMJ Open.

[63] Fluharty M., Taylor A.E., Grabski M., Munafò M.R. (2017). The Association of Cigarette Smoking With Depression and Anxiety: A Systematic Review. Nicotine Tob. Res..

[64] Paulus M.P., Stewart J.L. (2014). Interoception and Drug Addiction. Neuropharmacology.

[65] Spada M.M., Caselli G., Nikčević A.V., Wells A. (2015). Metacognition in Addictive Behaviors. Addict. Behav..

[66] Flaudias V., Heeren A., Brousse G., Maurage P. (2019). Toward a Triadic Approach to Craving in Addictive Disorders: The Metacognitive Hub Model. Harv. Rev. Psychiatry.

[67] Durazzo T., Meyerhoff D., Murray D. (2015). Comparison of Regional Brain Perfusion Levels in Chronically Smoking and Non-Smoking Adults. Int. J. Environ. Res. Public Health.

[68] Kaplan R.C., Baldoni P.L., Strizich G.M., Pérez-Stable E.J., Saccone N.L., Peralta C.A., Perreira K.M., Gellman M.D., Williams-Nguyen J.S., Rodriguez C.J. (2021). Current Smoking Raises Risk of Incident Hypertension: Hispanic Community Health Study–Study of Latinos. Am. J. Hypertens..

[69] Haight T.J., Bryan R.N., Erus G., Davatzikos C., Jacobs D.R., D’Esposito M., Lewis C.E., Launer L.J. (2015). Vascular Risk Factors, Cerebrovascular Reactivity, and the Default-Mode Brain Network. NeuroImage.

[70] Gray J.C., Thompson M., Bachman C., Owens M.M., Murphy M., Palmer R. (2020). Associations of Cigarette Smoking with Gray and White Matter in the UK Biobank. Neuropsychopharmacol.

[71] Sutherland M.T., Riedel M.C., Flannery J.S., Yanes J.A., Fox P.T., Stein E.A., Laird A.R. (2016). Chronic Cigarette Smoking Is Linked with Structural Alterations in Brain Regions Showing Acute Nicotinic Drug-Induced Functional Modulations. Behav. Brain Funct..

[72] Kim S.H., Yun C.-H., Lee S.-Y., Choi K., Kim M.B., Park H.-K. (2012). Age-Dependent Association between Cigarette Smoking on White Matter Hyperintensities. Neurol. Sci..

[73] Power M.C., Deal J.A., Sharrett A.R., Jack C.R., Knopman D., Mosley T.H., Gottesman R.F. (2015). Smoking and White Matter Hyperintensity Progression: The ARIC-MRI Study. Neurology.

[74] Zubieta J.-K., Heitzeg M.M., Xu Y., Koeppe R.A., Ni L., Guthrie S., Domino E.F. (2005). Regional Cerebral Blood Flow Responses to Smoking in Tobacco Smokers After Overnight Abstinence. Am. J. Psychiatry.

[75] Nomi J.S., Farrant K., Damaraju E., Rachakonda S., Calhoun V.D., Uddin L.Q. (2016). Dynamic Functional Network Connectivity Reveals Unique and Overlapping Profiles of Insula Subdivisions: Dynamic Connections of Insula Subdivisions. Hum. Brain Mapp..

