# Supplementary material for: Precision Preventive Medicine of Relapse in Smoking Cessation: Can MRI Inform the Search of Intermediate Phenotypes?
Source: Biology (Basel). 2021 Dec 27;11(1):35. doi: 10.3390/biology11010035 (PMC8773102; doi:10.3390/biology11010035)
Supplement: Supplementary file 1 [file biology-11-00035-s001.zip › biology-1498359-supplementary.pdf]

biology MDPI

Supplementary Materials

Precision Preventive Medicine of relapse in Smoking cessation: can MRI inform the search of intermediate phenotypes?

Yolaine Rabat, PhD; Sandra Chanraud, PhD; Majd Abdallah, PhD; Igor Sibon, MD PhD and Sylvie Berthoz, PhD.

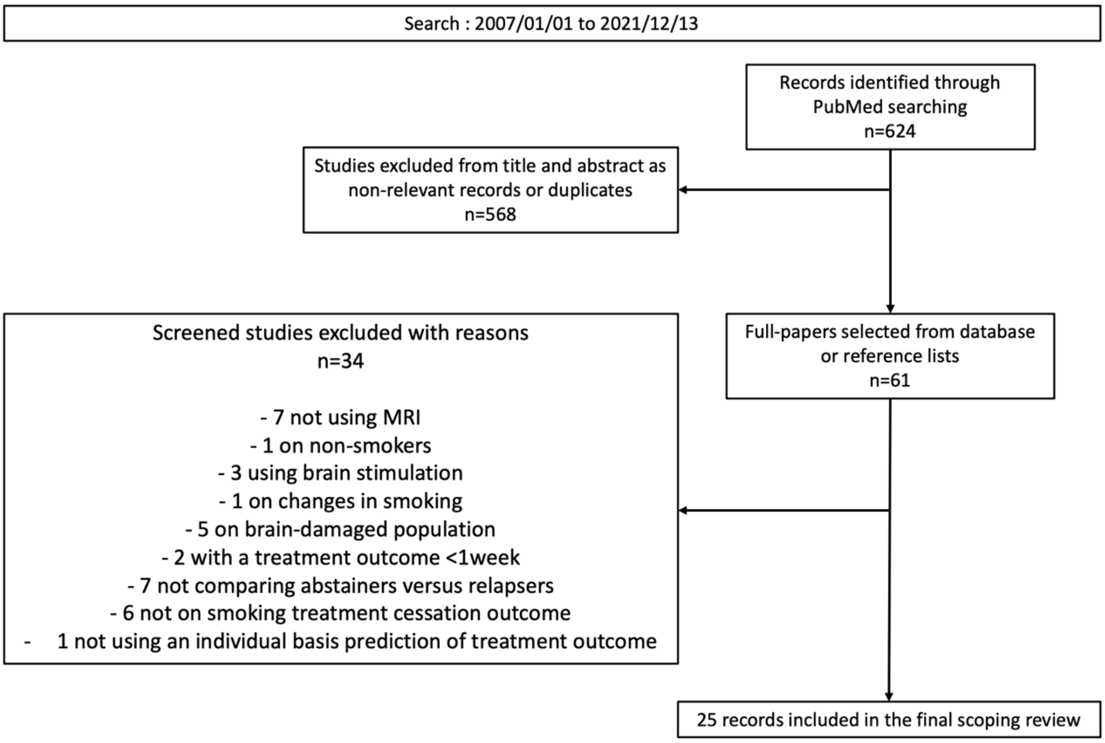

Figure S1. Study flow diagram.

The diagram displays the selection process of potentially relevant studies. Two authors (SB and YR) independently screened the title and abstracts of the 624 studies. Sixty-one potentially relevant full-text articles remained and were read independently by the two authors (SB and YR). They agreed to exclude 34 studies for specific reasons. Finally data extraction of the remaining 25 articles was undertaken by two authors (SB and YR) and checked by two additional authors (MA, SC).
